# Evaluation of carbapenem resistance using phenotypic and genotypic techniques in *Enterobacteriaceae* isolates

**DOI:** 10.1186/s12941-015-0105-1

**Published:** 2015-10-06

**Authors:** Kazım Sahin, Ayse Tekin, Sule Ozdas, Demet Akin, Hande Yapislar, Aziz Ramazan Dilek, Emine Sonmez

**Affiliations:** Department of Microbiology, Medical Faculty, Recep Tayyip Erdogan University, Islampasa mah., 53000 Rize, Turkey; Department of Infectious Diseases, Cengiz Gökcek Women and Obstetrics Hospital, Osmangazi mah., 27010 Gaziantep, Turkey; Department of Molecular Biology and Genetics, Medical Faculty, Istanbul Bilim University, Buyukdere cad., No: 120, Esentepe-Sisli-Istanbul, Turkey; Department of Pharmacology, Medical Faculty, Istanbul Bilim University, Buyukdere cad., No: 120, 34394 Esentepe-Sisli-Istanbul, Turkey; Department of Physiology, Medical Faculty, Istanbul Bilim University, Buyukdere cad., No: 120, 34394 Esentepe-Sisli-Istanbul, Turkey; Department of Infectious Diseases, Medical Faculty, Recep Tayyip Erdogan University, Islampasa mah., 53000 Rize, Turkey

**Keywords:** *Enterobacteriaceae*, Carbapenemase, Carbapenem resistance, OXA-48, NDM-1, Modified Hodge test, Metallo-β-lactamase antimicrobial gradient test

## Abstract

**Background:**

Bacterial resistance to antibiotics is increasing worldwide. Antibiotic-resistant strains can lead to serious problems regarding treatment of infection. Carbapenem antibiotics are the final treatment option for infections caused by serious and life-threatening multidrug-resistant gram-negative bacteria. Therefore, an understanding of carbapenem resistance is important for infection control. In the study described herein, the phenotypic and genotypic features of carbapenem-resistant *Enterobacteriaceae* strains isolated in our hospital were evaluated.

**Methods:**

In total, 43 carbapenem-resistant strains were included in this study. Sensitivity to antibiotics was determined using the VITEK^®^2 system. The modified Hodge test (MHT) and metallo-β-lactamase (MBL) antimicrobial gradient test were performed for phenotypic identification. Resistance genes IMP, VIM, KPC, NDM-1, and OXA-48 were amplified by multiplex PCR.

**Results:**

The OXA-48 gene was detected in seven strains, and the NDM-1 gene in one strain. No resistance genes were detected in the remainder of strains. A significant correlation was observed between the MHT test and OXA-48 positivity, and between the MBL antimicrobial gradient test and positivity for resistance genes (*p* < 0.05).

**Conclusion:**

The finding of one NDM-1-positive isolate in this study indicates that carbapenem resistance is spreading in Turkey. Carbapenem resistance spreads rapidly and causes challenges in treatment, and results in high mortality/morbidity rates. Therefore, is necessary to determine carbapenem resistance in Enterobacteriaceae isolates and to take essential infection control precautions to avoid spread of this resistance.

## Background

The increase in antibiotic resistance in the *Enterobacteriaceae* family has become a major threat to public health. Especially in recent years, *Enterobacteriaceae* family members have been identified as important nosocomial pathogens; infection can lead to severe morbidity and mortality, particularly in intensive care units (ICU), internal medicine and surgical units, and pediatric units [1].

Within the *Enterobacteriaceae* family, carbapenem-resistant *Klebsiella pneumoniae* strains have recently been noted in many parts of the world [2, 3]. Carbapenemase, an enzyme belonging to the *K. pneumoniae* carbapenemase (KPC) gene family, causes resistance by breaking down carbapenems. This enzyme was first isolated from a *K. pneumoniae* strain in 1996 and is included in the Ambler classification of β-lactamases. Although KPC β-lactamases are mostly found in *K. pneumoniae*, they can also be found in *Enterobacter* and *Salmonella* species. While metallo-β-lactamase (MBL) enzymes (which belong to Ambler class B) are frequently observed in non-fermentative bacteria, they can also be found in enteric bacteria. The most frequently described MBLs are Verona integron encoded metallo-*β*-*lactamase* (VIM), imipenem-hydrolyzing β-lactamase (IMI), Seoul imipenemase (SIM), and German imipenemase (GIM) enzymes.

The Clinical and Laboratory Standards Institute (CLSI) has increased the minimum inhibitory concentration (MIC) value or reduced the disk diffusion zones and recommended confirmation of carbapenemase production by the modified Hodge test (MHT) of *Enterobacteriaceae* in order to facilitate the detection of *Enterobacteriaceae* [4].

While resistance of chromosomal-mediated (intrinsic) carbapenemases is limited, some plasmid-mediated carbapenemases have emerged in recent years. These enzymes have primarily been described in *Klebsiella* spp. and *Serratia marcescens*, and throughout different countries [5, 6]. Plasmid-mediated (extrinsic) carbapenemases can hydrolyse other β-lactam antibiotics in addition to carbapenems. These are the members of Ambler class A, B and D β-lactamases. The SME, NMC, IMI, KPC, and GES enzymes comprise class A carbapenemases. Moreover, IPM, VIM, GIM, SPM, SIM, and NDM-1 enzymes comprise class B, and OXA enzymes that hydrolyse oxacillins comprise class D [7].

In Turkey, resistance mediated by KPC and New Delhi metallo-β-lactamase (NDM-1) enzymes in gram-negative enteric bacilli have been reported [8, 9]. Class D OXA carbapenemase-mediated carbapenem resistance has also been observed among gram-negative enteric bacilli in the context of nosocomial infections.

However, there are a limited number of studies related to carbapenem resistance mechanisms. In the study described herein, the characteristics of phenotypic and genotypic resistance of the carbapenem-resistant *Enterobacteriaceae* (*Escherichia coli* and *Klebsiella* spp.) strains isolated from patients in our hospital were evaluated. The clinical importance of this resistance was also determined.

## Methods

Our study was conducted on isolates derived from the following clinical samples: blood, rectal swab, urine, peritoneal fluid, sputum, tracheal aspirate, wounds, bronchoalveolar lavage (BAL) and pleural fluid, etc. Samples were isolated from patients in intensive care, internal intensive care, the surgical clinic, the internal medicine clinic, and the bone marrow transplantation (BMT) unit at Istanbul Bilim University Europe Florence Nightingale Hospital between January 7th 2012 to January 7th 2013, and subsequently transferred to the microbiology laboratory. The experiments were conducted on 43 carbapenem-resistant *Enterobactericeae* isolates. Only one strain from each patient was included in the study. *Enterobactericeae* strains were detected as carbapenem-resistant by using the VITEK^®^2 automated system (bioMérieux, France). Bacterial suspensions were stored in freezing tubes including beads at −40 °C. Sensitivities to IMP (imipenem), MEM (meropenem) and ERT (ertapenem) were confirmed by the antimicrobial gradient test in collected isolates. Carbapenemase production was determined by the MHT. Metallo-β-lactamase (MBL) production was evaluated using IMP and IMP/EDTA antimicrobial gradient test strips (E-test, bioMérieux, France). When the rate of IMP/IMP + EDTA was ≥8, isolates considered MBL-positive. When the rate of IMP/IMP + EDTA was <8, isolates were considered MBL-negative [10].

Multiplex PCR (Hyplex^®^ SuperBug ID, Germany) was used to search for genes affecting carbapenemase production. DNA extraction was conducted using the AxyPrep Microbial DNA Isolation Kit (Axygen Biosciences, USA) that lysed microorganisms by heating, use of detergents and mechanical techniques. The Hyplex^®^ SuperBug ID system was used to detect VIM, IPM, NDM-1, OXA-48, and KPC. No additional control strains were used.

Mean, standard deviation, minimum–maximum, rate, and frequency values were used to determine statistical significance. A kappa test was used for compliance analysis. The SPSS 22.0 program was used for statistical analyses.

## Results

A total of 43 patients were registered for the study, whose mean age was 53.8 (range 3–86). 28 (65.1 %) were male and 15 (34.9 %) male.

Of 43 strains, 38 were *K. pneumoniae* (88.4 %), four were *E. coli* (9.3 %), and one was *K. oxytoca* (2.3 %). According to antimicrobial gradient test and VITEK^®^2 test results, the greatest resistance to these 43 isolates was to ERT among the carbapenems (97.7 and 100 %, respectively), followed by MEM (93 and 90.7 % resistance, respectively) and IMP (79.1 and 88.4 % resistance, respectively).

Forty percent of these patients were followed up in the internal ICU, 36 % were followed up in the surgery ICU, 12 % were followed up in the BMT unit, and 12 % were followed up in the internal medicine unit. Of these 25 patients, bacterial growth was observed in blood cultures (eight patients), respiratory isolates (six), rectal swabs (five), urine cultures (three), wound cultures (two), and peritoneal fluid culture (one). Additionally, 19 of 43 patients had a history of stem cell-solid organ transplantation.

According to the MHT, of 43 strains, 35 (85 %) were carbapenemase-positive. According to MBL antimicrobial gradient test results, only two were MBL-positive (4.7 %).

Seven of the isolates (16.3 %) were positive for the OXA-48 gene, as determined by multiplex PCR. The NDM-1 gene was detected only in one strain (2.3 %). VIM, IPM and KPC resistance genes were not detected in any isolate (Table 1).

**Table 1:** Results of multiplex PCR for carbapenem-resistance genes in *Enterobacteriaceae* isolates

Genes	Positive		Negative	
	n	%	n	%
OXA-48	7	16.3	36	83.7
VIM	0	0.0	43	100.0
IPM	0	0.0	43	100.0
NDM-1	1	2.3	42	97.7
KPC	0	0.0	43	100.0

There was no significant correlation between OXA-48 resistance gene positivity and the ERT antimicrobial gradient test, MEM antimicrobial gradient test or IMP antimicrobial gradient test, nor was there a correlation between OXA-48 resistance gene positivity and ERT VITEK^®^2 or MEM VITEK^®^2 results (*p* > 0.05). Additionally, no correlation was detected between OXA-48 resistance gene positivity and IMP VITEK^®^2 results (Table 2).

**Table 2:** Relationship between susceptibility to carbapenems and OXA-48 gene positivity

Antibiotics	E-TEST and OXA-48					VITEK^®^ 2 and OXA-48				
	Positive		Negative		*p*	Positive		Negative		*p*
	S	R	S	R		S	R	S	R	
ERT	7	0	36	0	0.655	7	0	36	0	–
MEM	5	2	34	2	0.407	6	1	35	1	0.055
IMP	5	2	33	3	0.587	4	3	32	4	0.126

*ERT* ertapenem, *MEM* meropenem, *IMP* imipenem, *S* sensitive, *R* resistance

A correlation was observed between OXA-48 resistance gene positivity and MHT positivity (*p* = 0.004). A significant relationship between the MBL antimicrobial gradient test and NDM-1 was also observed (*p* = 0.000) (Table 3).

**Table 3:** Relationship between MHT and MBL E-test results with OXA-48 and NDM-1 genes positivity

Test	Positive		Negative		*p*
	n	%	n	%	
OXA-48					
MHT					
Positive	3	43	32	88.9	0.004
Negative	4	57	4	11.1	
NDM-1					
MBL E-test					
Positive	1	100	1	2.4	0.000
Negative	0	0	41	97.6	

*MHT* modifiye Hodge test, *MBL E-test* metallo-beta-lactamase E-test

## Discussion

Carbapenem antibiotics are the last treatment option for severe, life-threatening infections caused by gram negative enteric bacilli with multiple drug resistance. Therefore, an understanding of carbapenem resistance is important. The emergence of resistant strains, as well as those with the potential to become resistant, can differ according to geographical region.

The source of microorganisms leading to hospital outbreaks and determination of the transmission path, as well as the identification of control methods, create a basis for epidemiological studies. Rapid identification of strains is important whether or not there is an outbreak and provides crucial information regarding the control of hospital outbreaks. Antibiotic susceptibility tests have become fundamental tools in the identification of many hospital outbreaks due to their common usage. Moreover, these tests are standardized and can be applied to many other factors. However, as for other phenotypic methods, antibiotic susceptibility profiles are not ideal because they can be affected by growth medium, phase of reproduction and rate of spontaneous mutations. Therefore, genotyping techniques are preferentially used worldwide. With regard to determining the relationship between bacterial isolates, the techniques of serotyping, biotyping, antibiotyping, and bacteriophage typing have been replaced by others, including plasmid analysis and PCR-based typing methods, such as ribotyping [11].

In Turkey, molecular studies focusing on carbapenem resistance mechanisms are limited [8, 12]. Some regional studies have shown an association between the OXA-48 gene and carbapenem resistance in gram-negative enteric bacilli [13, 14]. It was recently reported that carbapenem resistance may be associated with certain situations, such as being transferred between hospitals or from long-term care centers, stay in the ICU, the presence of a central venous catheter, usage of antibiotics, and having diabetes mellitus [15, 16]. In this study, 27 of 43 patients were monitored in the ICU. Since our hospital was the reference hospital for units such as cardiovascular surgery and transplantation of stem cells and solid organs, most of our patients had follow-up or hospitalization histories prior to being admitted to our hospital.

In our study, of 43 patients infected with carbapenem-resistant *Enterobacteriaceae* strains, 25 patients died (58.1 %). Patel et al. [2] demonstrated that the mortality rates of carbapenem-resistant *K. pneumoniae* infected in-patients were significantly higher compared to patients infected with carbapenem-sensitive strains (48 and 20 %, respectively; *p* = 0.001). In this study, it was noted that receiving a transplant was an independent factor in carbapenem-resistant *K. pneumoniae* infection. In another study, the mortality rate was 60 % in a heterogeneous patient population that included carbapenem-resistant *K. Pneumoniae*-infected transplant recipients [17]. In addition, staying in the ICU, surgical procedures, using catheter, length of hospitalization and using of cephalosporins and aminoglycosides are risk factors for carbapenem-resistant *K. pneumoniae* infections [18, 19].

In our study, sensitivity was determined using the automated VITEK^®^2 Compact system and the antimicrobial gradient test. Of the 43 strains, all were ertapenem-resistant, 95.3 % were meropenem-resistant and 83.7 % were imipenem-resistant. A resistance rate of 97.7 % to ertapenem was detected using antimicrobial gradient test techniques, whereas the resistance rate was 100 % according to the VITEK^®^2 automated system. Additionally, meropenem resistance was calculated using the antimicrobial gradient test (93 %) and the automated system (90.7 %).

The MHT is one of the simplest techniques used to indicate carbapenemase activity. In this test, carbapenem inhibition zone diameter and MIC values are considered sufficient [4] and valuable for the determination of KPC, OXA and MBL enzymes in laboratories in which dilution techniques cannot be performed [2, 20]. Herein, a correlation was found between MHT technique results and OXA-48 gene positivity (*p* = 0.004) (Table 3). Raghunathan et al. conducted studies in which they determined the carbapenem resistance of *Enterobacteriaceae* strains by comparing PCR and MHT techniques. The authors revealed a 96 % correlation between PCR positivity and MHT positivity in terms of KPC genes (causing carbapenem resistance) [21]. MHT is not sufficient to detect OXA-48 or other carbapenemase producing isolates. Combining carbapenemase inhibitor test, using phenylboronic acid, with MHT is a good indicator fort the detection of carbapenemase producing isolates [22].

In the present study, the MBL antimicrobial gradient test was used to detect class B metallo-β-lactamases. Of the 43 isolates, MBL positivity was found in two. Of these two isolates, only one was NDM-1 positive according to PCR results. A correlation was observed between MBL antimicrobial gradient test results and NDM-1 positivity/negativity by multiplex PCR (Table 3). NDM-1 resistance has been described in several case reports in recent years [14, 23]. However, class D OXA carbapenemase-mediated carbapenem resistance has become prevalent, especially among *Enterobacteriaceae* strains causing nosocomial infections. In our country, an OXA-48 type β-lactamase belonging to class D carbapenemases was detected for the first time at the Istanbul University Faculty of Medicine Hospital. This carbapenemase enzyme was isolated from *K. pneumoniae* strains obtained from a urine culture of an in-patient [24]. The OXA-48 carbapenemase enzyme has mostly been identified in *K. pneumoniae* strains in Turkey, Lebanon and Belgium [14, 25, 26]. Two studies evaluated carbapenem resistance in ESBL-producing carbapenem-resistant *K. pneumoniae* strains using disc diffusion and antimicrobial gradient test techniques [27, 28]. Of the 14 strains examined, the OXA-1 gene was detected in all, the OXA-48 gene in two, and the NDM-1 gene in two [27]. In addition, the IS1999 location was shown in strains expressing OXA-48, indicating that ERT resistance was higher in OXA-48 positive strains compared to other carbapenems [29]. According to studies performed in Greece and Spain, all carbapenem-resistant *K. pneumoniae* strains were positive for the OXA-48 gene. The OXA-48 gene was associated with the IS1999 element [30, 31]. In Turkey, the first NDM-1 resistance gene was identified in 2011 by Poirel et al. [14]. This gene was isolated from an allogeneic stem cell-transplanted leukemia patient from Iraq. From this isolated case, NDM-1 was identified as the first resistance gene detected from an international origin [14]. Yanik et al. [12] conducted a study in Samsun-Turkey in which the carbapenemase enzyme was detected using the MHT technique in gram-negative strains resistant to at least one of the antibiotics (imipenem, meropenem or doripenem). PCR was performed to detect NDM-1, but no NDM-1 gene resistance was found in any of the 210 clinical isolates. These results indicate that NDM-1 is not yet common in Turkey. The NDM-1 resistance gene with no history of foreign contacts was firstly determined by Alp et al. [23], in which 137 carbapenem-resistant *K. pneumoniae* strains were examined for resistance genes. Of 127 strains, OXA-48 was detected in 91.5 %, IMP in 3.2 %, NDM-1 in 4.3 %, and both OXA-48 and NDM-1 in 1 %. This study described herein has added Turkey to the list of countries in which NDM-1 has been isolated. According to our results, *K. pneumoniae* isolates were found to be NDM-1-positive (Table 3). This *K. pneumoniae* was isolated from a rectal swab of a patient who did not have any foreign origin.

It has been reported that carbapenem-producing *Enterobactericeae* strains lead to both infection and colonisation, and these microorganisms cause nosocomial outbreaks as well as epidemics. Additionally, it is known that OXA carbapenemase-mediated carbapenem resistance in particular is increasing [20]. Therefore, if there are high MIC values against carbapenems in *Klebsiella* strains, other accompanying resistance mechanisms should also be examined. In addition to phenotypic tests, genotypic investigations should be commonly used in order to decrease mortality and morbidity due to infections with *Enteobactericeae* spp.

## Conclusion

The finding of one NDM-1-positive isolate in this study indicates that carbapenem resistance is spreading in Turkey. The finding of one NDM-1-positive isolate in this study indicates that carbapenem resistance is spreading in Turkey. Carbapenem resistance spreads rapidly and causes challenges in treatment, and results in high mortality/morbidity rates. Therefore, is necessary to determine carbapenem resistance in *Enterobacteriaceae* isolates and to take essential infection control precautions to avoid spread of this resistance.

## References

[1] Nordmann P, Naas T, Poirel L (2011). Global spread of carbapenemase-producing *Enterobacteriaceae*. Emerg Infect Dis.

[2] Patel JB, Rasheed JK, Kitchel B (2009). Carbapenemases in *Enterobacteriaceae*: activity, epidemiology, and laboratory detection. Clin Microbiol Newsl.

[3] Shah PM (2008). Parenteral carbapenems. Clin Microbiol Infect.

[4] Performance standards for antimicrobial susceptibility testing, 21st informational supplement. Clinical and Laboratory Standards Institute; 2013.

[5] Tsakris A, Kristo I, Poulou A, Markou F, Ikonomidis A, Pournaras S (2008). First occurrence of KPC-2-possessing *Klebsiella pneumoniae* in a Greek hospital and recommendation for detection with boronic acid disc tests. J Antimicrob Chemother.

[6] Wei ZQ, Du XX, Yu YS, Shen P, Chen YG, Li LJ (2007). Plasmid-mediated KPC-2 in a *K. pneumoniae* isolate from China. Antimicrob Agents Chemother.

[7] Queenan AM, Bush K. Carbapenemases: the versatile beta-lactamases. Clin Microbiol Rev. 2007;20:440–58 **(table of contents)**. 10.1128/CMR.00001-07 PMC1932750 17630334

[8] Gür D, Hasçelik G, Aydın N, Telli M, Gültekin M, Ogul D (2009). Antimicrobial resistance in gram-negative hospital isolates: results of the Turkish HITIT-2 Surveillance Study of 2007. J Chemother.

[9] Korten V, Ulusoy S, Zarakolu P, Mete B (2007). Antibiotic resistance surveillance over a 4-year period (2000–2003) in Turkey: results of the MYSTIC Program. Diagn Microbiol Infect Dis.

[10] Yan JJ, Wu JJ, Tsai SH, Chuang CL (2004). Comparison of the double-disk, combined disk, and E test methods for detecting metallo-beta-lactamases in gram-negative bacilli. Diagn Microbiol Infect Dis.

[11] Us E, Tekeli A, Arıkan Akan O, Dolapçı I, Şahin F, Karahan ZC (2010). Molecular epidemiology of carbapenem-resistant *Klebsiella pneumoniae* strains isolated between 2004–2007 in Ankara University Hospital, Turkey. Mikrobiyol Bul.

[12] Yanık K, Emir D, Eroglu C, Karadag A, Guney AK, Gunaydin M (2013). Investigation of the presence of New Delhi metallo-beta-lactamase-1 (NDM-1) by PCR in carbapenem-resistant gram-negative isolates. Mikrobiyol Bul.

[13] Aktas Z, Kayacan CB, Schneider I, Can B, Midilli K, Bauernfeind A (2008). Carbapenem-hydrolyzing oxacillinase, OXA-48, persists in *Klebsiella pneumoniae* in Istanbul, Turkey. Chemotherapy.

[14] Poirel L, Özdamar M, Ocampo-Sosa AA, Türkoğlu S, Ozer UG, Nordmann P (2012). NDM-1-producing *Klebsiella pneumoniae* now in Turkey. Antimicrob Agents Chemother.

[15] Perez F, Van Duin D (2013). Carbapenem-resistant *Enterobacteriaceae*: a menace to our most vulnerable patients. Cleve Clin J Med.

[16] Saidel-Odes L, Borer A (2013). Limiting and controlling carbapenem-resistant. Infect Drug Resist.

[17] Nguyen M, Eschenauer GA, Bryan M, O’Neil K, Furuya EY, Della-Latta P (2010). Carbapenem-resistant *Klebsiella pneumoniae* bacteremia: factors correlated with clinical and microbiologic outcomes. Diagn Microbiol Infect Dis.

[18] Dizbay M, Tunccan OG, Karasahin O, Aktas F (2014). Emergence of carbapenem-resistant *Klebsiella* spp infections in a Turkish university hospital: epidemiology and risk factors. J Infect Dev Ctries.

[19] Teo J, Cai Y, Tang S, Lee W, Tan TY, Tan TT (2012). Risk factors, molecular epidemiology and outcomes of ertapenem-resistant, carbapenem-susceptible *Enterobacteriaceae*: a case–case–control study. PLoS One.

[20] Yigit H, Queenan AM, Rasheed JK, Biddle JW, Domenech-Sanchez A, Alberti S (2003). Carbapenem-resistant strain of *Klebsiella oxytoca* harboring carbapenem-hydrolyzing beta-lactamase KPC-2. Antimicrob Agents Chemother.

[21] Raghunathan A, Samuel L, Tibbetts RJ (2011). Evaluation of a real-time PCR assay for the detection of the *Klebsiella pneumoniae* carbapenemase genes in microbiological samples in comparison with the modified Hodge test. Am J Clin Pathol.

[22] Song W, Hong SG, Yong D, Jeong SH, Kim HS (2015). Combined use of the modified Hodge test and carbapenemase inhibition test for detection of carbapenemase-producing *Enterobacteriaceae* and metallo-β-lactamase-producing *Pseudomonas* spp. Ann Lab Med.

[23] Alp E, Percin D, Colakoglu S, Durmaz S, Kurkcu CA, Ekincioglu P (2013). Molecular characterization of carbapenem-resistant *Klebsiella pneumoniae* in a tertiary university hospital in Turkey. J Hosp Infect.

[24] Carrer A, Poirel L, Yılmaz M, Akan OA, Feriha C, Cuzon G (2010). Spread of OXA-48 encoding plasmid in Turkey and beyond. Antimicrob Agents Chemother.

[25] Cuzon G, Naas T, Bogaerts P, Glupczynski Y, Huang TD, Nordmann P (2008). Plasmid-encoded carbapenem hydrolyzing beta-lactamase OXA-48 in an imipenem susceptible *Klebsiella pneumoniae* strain from Belgium. Antimicrob Agents Chemother.

[26] Matar GM, Cuzon G, Araj GF, Naas T, Corkill J, Kattar MM (2008). Oxacillinase-mediated resistance to carbapenems in *Klebsiella pneumoniae* from Lebanon. Clin Microbiol Infect.

[27] Baroud M, Dandache I, Araj GF, Wakim R, Kanj S, Kanafani Z (2013). Underlying mechanisms of carbapenem resistance in extended spectrum beta-lactamase producing *Klebsiella pneumoniae and Escherichia coli* isolates at a tertiary care centre in Lebanon: role of OXA-48 and NDM-1 carbapenemases. Int J Antimicrob Agents.

[28] Eser Koseoglu O, Uludag Altun H, Ergin A, Boral B, Sener B, Hascelik G (2014). Carbapenem resistance in ESBL positive *Enterobacteriaceae* isolates causing invasive infections. Mikrobiyol Bul.

[29] Voulgari E, Zarkotou O, Ranellou K, Karageorgopoulos DE, Vrioni G, Mamali V (2013). Outbreak of OXA-48 carbapenemase-producing *Klebsiella pneumoniae* in Greece involving an ST11 clone. J Antimicrob Chemother.

[30] Pano-Pardo JR, Ruiz-Carrascoso G, Navarro-San Francisco C, Gomez-Gil R, Mora- Rillo M (2013). Infections caused by OXA-48-producing *Klebsiella pneumoniae* in a tertiary hospital in Spain in the setting of a prolonged, hospital-wide outbreak. J Antimicrob Chemother.

[31] Adler A, Shklyar M, Schwaber MJ, Navon-Venezia S, Dhaher Y, Edgar R (2011). Introduction of OXA-48-producing *Enterobacteriaceae* to Israeli hospitals by medical tourism. J Antimicrob Chemother.

